# Supplementary material for: MR perfusion source mapping depicts venous territories and reveals perfusion modulation during neural activation
Source: Nat Commun. 2025 Apr 24;16:3890. doi: 10.1038/s41467-025-59108-3 (PMC12022259; doi:10.1038/s41467-025-59108-3)
Supplement: Supplementary file 1 — Supplementary Information [file 41467_2025_59108_MOESM1_ESM.pdf]

# Supplementary Information

MR Perfusion Source Mapping Depicts Venous Territories and Reveals Perfusion Modulation during Neural Activation

This supplementary information document contains two additional figures: Supplementary Figure 1 demonstrating our method's ability to detect remote sources from deep cerebral veins and Supplementary Figure 2 illustrating local perfusion modulation due to neural activation in a third subject.

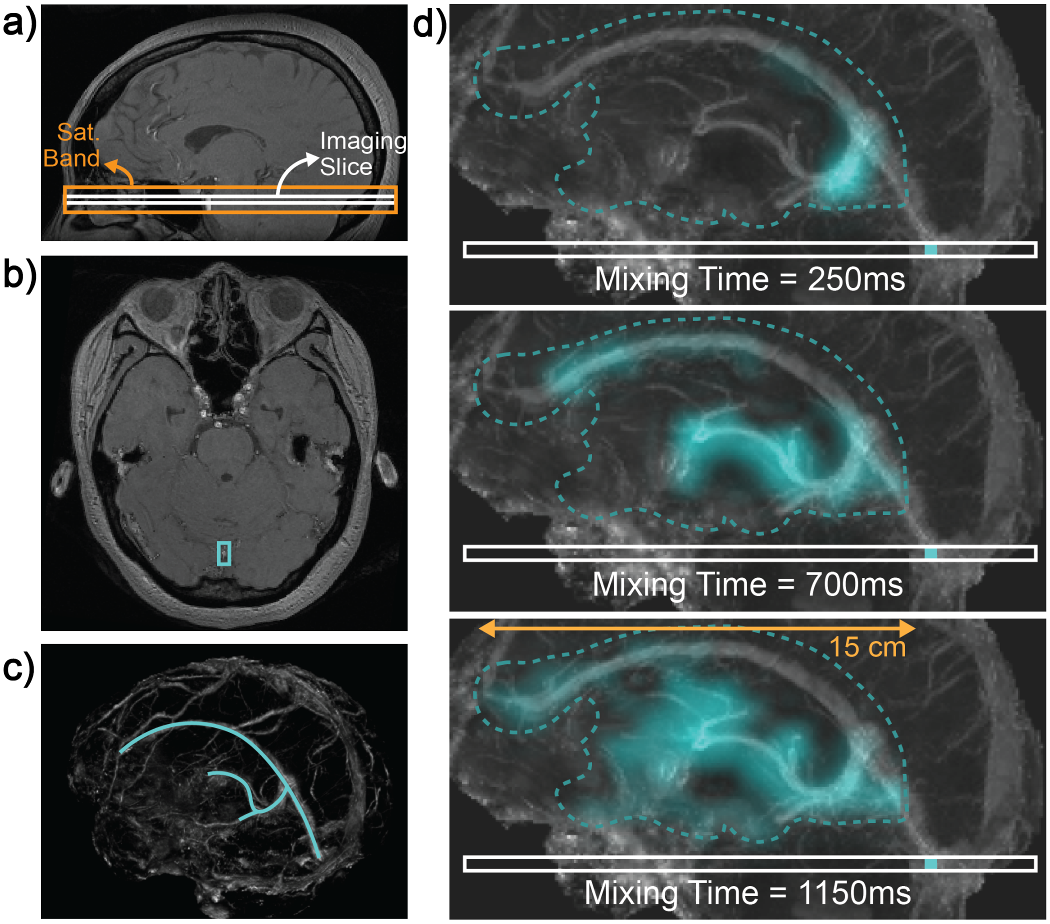


**Supplementary Figure 1** : Experimental setup and results for perfusion source mapping of the deep cerebral veins. a) A sagittal image showing the placement of the axial imaging slice and the saturation band. b) ROI selected on the straight sinus. c) The deep cerebral veins marked on the 3D QSM-venogram. d) Perfusion source maps of the selected ROI at mixing times of 250, 700, and 1150 ms overlaid on the QSM-venogram of the sagittal section containing the deep cerebral veins. The venous territory drained by the straight sinus is delineated with dashed contours. The perfusion source maps demonstrate the ability to resolve sources originating near the center of the brain. The sources are detected up to 15 cm away from the selected ROI, indicated by the orange double-headed arrow.

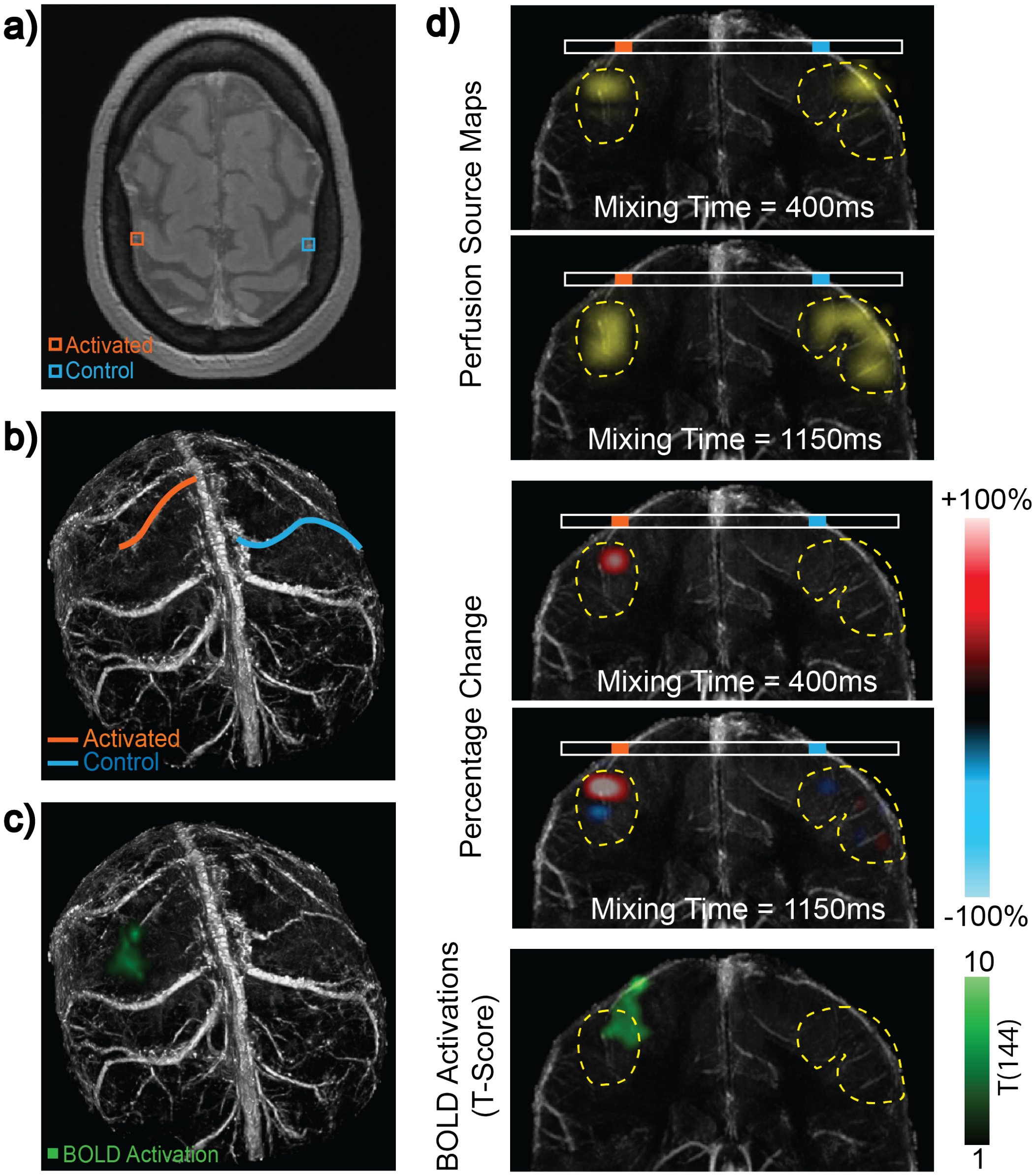


**Supplementary Figure 2** : Right motor cortex activation in Subject 3. a) Imaging slice showing two selected veins: one (orange) drains the activated right motor cortex and one (blue) is selected as control. b) The color-coded veins overlaid on a 3D QSM-venogram. c) BOLD activations (green) overlaid on the 3D QSM-venogram. d) Top: Perfusion source maps for activated and control veins at two mixing times. Middle: The percentage change between task and baseline perfusion source maps showing mostly positive local percentage change near the activated regions. Little or no percentage change is observed in the control vein. Bottom: BOLD T-scores for the coronal sections containing each vein. One-sided t-tests were conducted to obtain t-statistic images with a cluster significance threshold of $p = 0.01$ and a cluster extent threshold of 10. Venous territories are marked with yellow dashed contours.
