# Supplementary material for: MR perfusion source mapping depicts venous territories and reveals perfusion modulation during neural activation
Source: Nat Commun. 2025 Apr 24;16:3890. doi: 10.1038/s41467-025-59108-3 (PMC12022259; doi:10.1038/s41467-025-59108-3)
Supplement: Supplementary file 2 — Reporting Summary [file 41467_2025_59108_MOESM2_ESM.pdf]

nature portfolio

Corresponding author(s): Ekin Karasan

Last updated by author(s): 3/29/2025

Reporting Summary

Nature Portfolio wishes to improve the reproducibility of the work that we publish. This form provides structure for consistency and transparency in reporting. For further information on Nature Portfolio policies, see our Editorial Policies and the Editorial Policy Checklist.

Statistics

For all statistical analyses, confirm that the following items are present in the figure legend, table legend, main text, or Methods section.

n/a	Confirmed	
☐	☒	The exact sample size (*n*) for each experimental group/condition, given as a discrete number and unit of measurement
☐	☒	A statement on whether measurements were taken from distinct samples or whether the same sample was measured repeatedly
☐	☒	The statistical test(s) used AND whether they are one- or two-sided<br>*Only common tests should be described solely by name; describe more complex techniques in the Methods section.*
☒	☐	A description of all covariates tested
☒	☐	A description of any assumptions or corrections, such as tests of normality and adjustment for multiple comparisons
☒	☐	A full description of the statistical parameters including central tendency (e.g. means) or other basic estimates (e.g. regression coefficient) AND variation (e.g. standard deviation) or associated estimates of uncertainty (e.g. confidence intervals)
☐	☒	For null hypothesis testing, the test statistic (e.g. *F*, *t*, *r*) with confidence intervals, effect sizes, degrees of freedom and *P* value noted<br>*Give P values as exact values whenever suitable.*
☒	☐	For Bayesian analysis, information on the choice of priors and Markov chain Monte Carlo settings
☒	☐	For hierarchical and complex designs, identification of the appropriate level for tests and full reporting of outcomes
☒	☐	Estimates of effect sizes (e.g. Cohen's *d*, Pearson's *r*), indicating how they were calculated

Our web collection on statistics for biologists contains articles on many of the points above.

Software and code

Policy information about availability of computer code

Data collection	Data collection was performed with an MRI pulse sequence implemented on a proprietary software tool RTHawk (Vista AI, Palo Alto, CA).
Data analysis	The code needed used for data processing can be found in the public repository (https://github.com/mikgroup/Venous-Perfusion-Source-Mapping). We used STI Suite V3.0 (https://people.eecs.berkeley.edu/~chunlei.liu/software.html) to obtain susceptibility maps.

For manuscripts utilizing custom algorithms or software that are central to the research but not yet described in published literature, software must be made available to editors and reviewers. We strongly encourage code deposition in a community repository (e.g. GitHub). See the Nature Portfolio guidelines for submitting code & software for further information.

Data

Policy information about availability of data

All manuscripts must include a data availability statement. This statement should provide the following information, where applicable:

- Accession codes, unique identifiers, or web links for publicly available datasets
- A description of any restrictions on data availability
- For clinical datasets or third party data, please ensure that the statement adheres to our policy

All main data used and analyzed in this study are openly available in the Zenodo repository at DOI: 10.5281/zenodo.15041913

Research involving human participants, their data, or biological material

Policy information about studies with human participants or human data. See also policy information about sex, gender (identity/presentation), and sexual orientation and race, ethnicity and racism.

Reporting on sex and gender	The data collected in this manuscript was acquired from three male and two female subjects, with sex determined through self-reporting. Sex was not considered as a variable in this study because the research was focused on demonstrating the method. Statistical analysis was only performed between datasets acquired from the same subject, without comparisons between different subjects. No sex- or gender-based analysis was conducted, as this was not relevant to the study's aims.
Reporting on race, ethnicity, or other socially relevant groupings	No socially constructed or socially relevant categorization variables were used in this manuscript, and no analyses were performed on such variables. There were no classifications or controls for confounding variables related to these categories.
Population characteristics	There are no covariate-relevant population characteristics in this study.
Recruitment	The participants were recruited from within the lab group. A potential source of bias may stem from the fact that all participants work in a similar field. However, as this research focuses on method demonstration rather than generalizable outcomes across a wider population, the impact of this bias on the study's findings should be minimal.
Ethics oversight	Data collection was approved by the Institutional Review Board at UC Berkeley (CPHS #2010-07-1830). Written consent was obtained from all subjects.

Note that full information on the approval of the study protocol must also be provided in the manuscript.

Field-specific reporting

Please select the one below that is the best fit for your research. If you are not sure, read the appropriate sections before making your selection.

☒ Life sciences ☐ Behavioural & social sciences ☐ Ecological, evolutionary & environmental sciences

For a reference copy of the document with all sections, see nature.com/documents/nr-reporting-summary-flat.pdf

Life sciences study design

All studies must disclose on these points even when the disclosure is negative.

Sample size	The sample size for this study was five subjects. No formal sample size calculation was performed, as the primary objective was to demonstrate the capabilities of the proposed imaging method rather than to conduct population-level statistical analysis. Given the methodological nature of the study, this sample size was considered sufficient to validate the approach.
Data exclusions	No data were excluded from the analyses.
Replication	To verify the reproducibility of our findings, we repeated the experiments measuring blood flow changes during neural activation twice in two subjects. The results were consistent across repetitions.
Randomization	This was not relevant to the study, as no group analysis was performed.
Blinding	This was not relevant to the study, as no group analysis was performed.

Reporting for specific materials, systems and methods

We require information from authors about some types of materials, experimental systems and methods used in many studies. Here, indicate whether each material, system or method listed is relevant to your study. If you are not sure if a list item applies to your research, read the appropriate section before selecting a response.

Materials & experimental systems

n/a	Involved in the study	
☒	☐	Antibodies
☒	☐	Eukaryotic cell lines
☒	☐	Palaeontology and archaeology
☒	☐	Animals and other organisms
☒	☐	Clinical data
☒	☐	Dual use research of concern
☒	☐	Plants

Methods

n/a	Involved in the study	
☒	☐	ChIP-seq
☒	☐	Flow cytometry
☐	☒	MRI-based neuroimaging

Plants

Seed stocks

Report on the source of all seed stocks or other plant material used. If applicable, state the seed stock centre and catalogue number. If plant specimens were collected from the field, describe the collection location, date and sampling procedures.

Novel plant genotypes

Describe the methods by which all novel plant genotypes were produced. This includes those generated by transgenic approaches, gene editing, chemical/radiation-based mutagenesis and hybridization. For transgenic lines, describe the transformation method, the number of independent lines analyzed and the generation upon which experiments were performed. For gene-edited lines, describe the editor used, the endogenous sequence targeted for editing, the targeting guide RNA sequence (if applicable) and how the editor was applied.

Authentication

Describe any authentication procedures for each seed stock used or novel genotype generated. Describe any experiments used to assess the effect of a mutation and, where applicable, how potential secondary effects (e.g. second site T-DNA insertions, mosiacism, off-target gene editing) were examined.

Magnetic resonance imaging

Experimental design

Design type

Both task (fMRI) and resting-state (perfusion) measurements were made.

Design specifications

For task studies, 140 blocks were measured for each subject. The duration of each block was around 30 seconds and a delay of 2s was used between blocks.

Behavioral performance measures

No behavioral performance measures were recorded.

Acquisition

Imaging type(s)

Functional, perfusion (DiSpect)

Field strength

3

Sequence & imaging parameters

EPI BOLD fMRI: 2D EPI-BOLD sequence was performed with 29 slices, resolution= 3.3x3.3mm2, matrix=64x64, TE=28ms and TR=2s.
Spiral BOLD fMRI: 2D Spiral-BOLD sequence was performed with 29 slices, resolution= 4x4mm2, matrix=53x53, TE=28ms and TR=2s.
QSM: Multi-Echo 3D-GRE sequence was performed with 16 echo times, spatial resolution = 0.8x0.8x0.8mm3, TE1=2.12ms, ΔTE=2.42ms, TR=41.0 ms, FOV=21x21x13cm3, and Flip Angle=12degrees.
DiSpect: resolution = 4x4mm2, matrix = 40x40, flip angle= 90 degrees, TE/TR = 1.8ms/3s and slice thickness = 5mm.
Multi-Slice DiSpect: resolution = 4x4mm2, matrix = 53x53, flip angle= 90 degrees, TE/TR = 1.8ms/3s and slice thickness = 4mm.

Area of acquisition

For EPI BOLD fMRI, QSM and Spiral BOLD fMRI a whole brain acquisition was performed. With DiSpect, a single slice intersecting several large superior veins was selected to image their perfusion sources. For the DiSpect experiment imaging the deep cerebral veins a lower slice intersecting the straight sinus was prescribed. For the multi-slice DiSpect experiment three slices were prescribed towards the top of the head.

Diffusion MRI ☐ Used ☒ Not used

Preprocessing

Preprocessing software

The fMRI data processing was perform using SPM12 (https://www.fil.ion.ucl.ac.uk/spm/). The preprocessing steps included realigning, slice-timing correction, co-registration and smoothing (kernel size = 3x3x3mm3).

Normalization

Normalization was not performed.

Normalization template

Normalization was not performed.

Noise and artifact removal

Realigning and slice-timing corrections were performed as the first steps of preprocessing.

Volume censoring

Volume censoring was not performed.

Statistical modeling & inference

Model type and settings

For the statistical analysis of fMRI acquisitions, a mass univariate analysis was performed with a cluster significance threshold of p=0.01. For the first level model, the timings of the motor cortex activation task were entered into a design matrix.

Effect(s) tested

The contrast of interest was the difference between a motor cortex activation task compared to rest or baseline condition. ANOVA or factorial designs were not used.

Specify type of analysis: ☒ Whole brain ☐ ROI-based ☐ Both

Statistic type for inference
(See Eklund et al. 2016)

Cluster-wise analysis was performed with a cluster significance threshold of p=0.01 and a cluster extent threshold of 10.

Correction

Correction was not performed.

Models & analysis

n/a	Involved in the study
☒	☐ Functional and/or effective connectivity
☒	☐ Graph analysis
☒	☐ Multivariate modeling or predictive analysis
